# Effects of Exposure to 0.06 ppm Ozone on FEV_1_ in Humans: A Secondary Analysis of Existing Data

**DOI:** 10.1289/ehp.11396

**Published:** 2008-04-21

**Authors:** James S. Brown, Thomas F. Bateson, William F. McDonnell

**Affiliations:** 1 National Center for Environmental Assessment, U.S. Environmental Protection Agency, Research Triangle Park, North Carolina, USA; 2 National Center for Environmental Assessment, U.S. Environmental Protection Agency, Washington, DC, USA; 3 William F. McDonnell Consulting, Chapel Hill, North Carolina, USA

**Keywords:** air pollutants, photochemical oxidants, spirometry

## Abstract

**Background:**

Ozone is a potent photochemical oxidant that produces transient, reversible decrements in the lung function of acutely exposed individuals. A recent study provided previously unavailable clinical data for 30 healthy young adults exposed to O_3_ at 0.06 ppm. That study showed significant effects of 0.08 ppm on lung function, confirming the findings of others. However, exposure to 0.06 ppm O_3_ was not reported to significantly affect lung function.

**Objectives:**

We conducted this analysis to reevaluate the existing lung function data of the volunteers previously exposed to 0.06 ppm O_3_.

**Methods:**

We obtained pre- and postexposure data on forced expiratory volume in 1 sec (FEV_1_) for all subjects who were previously exposed for 6.6 hr to filtered air or to 0.06 ppm or 0.08 ppm O_3_. We used standard statistical methods appropriate for paired comparisons to reanalyze FEV_1_ responses after exposure to 0.06 ppm O_3_ relative to filtered air.

**Results:**

Controlling for filtered air responses, 24 of the 30 subjects experienced an O_3_-induced decrement in FEV_1_. On average, 0.06 ppm O_3_ exposure caused a 2.85% reduction in FEV_1_ (*p* < 0.002), which was consistent with the predicted FEV_1_ response from existing models. Although the average response was small, two subjects had > 10% FEV_1_ decrements.

**Conclusions:**

Exposure to 0.06 ppm O_3_ causes a biologically small but highly statistically significant decrease in mean FEV_1_ responses of young healthy adults.

Acute exposure to ozone causes transient respiratory symptoms, reversible decrements in pulmonary function, and an inflammatory response that may persist for at least 18–24 hr after exposure. A thorough review of recent epidemiologic and controlled human exposure studies is available elsewhere [U.S. Environmental Protection Agency (EPA) 2006]. Controlled exposures of healthy young adults show that the magnitude of these respiratory effects is a function of O_3_ concentration, minute ventilation (V_E_), and exposure duration. The primary focus of this article is the effect of short-term controlled O_3_ exposures on forced expiratory volume in 1 sec (FEV_1_) as a measure of lung function in healthy young adults. Because small changes in spirometry in healthy young adults may result from exercise, diurnal, or other effects in addition to the effects of O_3_ during the course of an exposure, we use the term “O_3_-induced” here to designate effects that we have corrected for such extraneous responses as measured during filtered air (FA) exposures.

After prolonged, 6.6-hr exposures to O_3_ at concentrations of ≥ 0.08 ppm, young healthy adults develop significant reversible decrements in FEV_1_ at a moderate level of exercise (V_E_ = 40 L/min). Exposures to 0.04-ppm O_3_ result in small, statistically nonsignificant O_3_-induced responses (Adams 2002). Volunteers exposed to 0.08 ppm O_3_ experience group mean O_3_-induced FEV_1_ decrements that range from 6% to 8% (Adams 2003, 2006; Horstman et al. 1990; McDonnell et al. 1991); those exposed to 0.10 ppm have group mean decrements of 8–14% (Horstman et al. 1990; McDonnell et al. 1991); and those exposed to 0.12 ppm have group mean decrements of 13–16% (Adams 2002; Folinsbee et al. 1988; Horstman et al. 1990). The distribution of individual responses becomes skewed with increasing exposure concentration and magnitude of the group mean FEV_1_ response (McDonnell 1996). Taken together, these data indicate that FEV_1_ responses are clearly affected by 6.6-hr exposures to O_3_ concentrations ≥ 0.08 ppm. These data also suggest that smaller, but measurable, responses are likely to occur with 6.6-hr exposure to concentrations somewhat below 0.08 ppm. We are particularly interested in estimating the magnitude of FEV_1_ responses to concentrations < 0.08 ppm, with the ultimate goals of better characterizing the concentration–response (C–R) relationship and reducing uncertainty in the assessment of risk at low O_3_ concentrations.

Until recently, published data have not been available for 6.6-hr exposures to O_3_ concentrations between 0.04 and 0.08 ppm. Adams (2006) provided results for healthy young individuals (15 males, 15 females) randomly exposed for 6.6 hr on six separate occasions to FA, to constant or square-wave (S-W) 0.06 ppm and 0.08 ppm O_3_, and to three variable concentration patterns having mean overall exposure O_3_ concentrations of 0.04, 0.06, and 0.08 ppm. The subjects (mean age ~ 23 years) were recruited from the University of California, Davis, and the surrounding community. Measures of FEV_1_ were conducted before exposure and after approximately 1, 2, 3, 4.6, 5.6, and 6.6 hr of exposure. Each 6.6-hr exposure was conducted in an exposure chamber and included six 1-hr periods in which participants alternated 50 min of exercise with 10 min of rest. An additional 35-min rest/lunch period in the chamber followed the third hour of exposure. Exercise was calibrated to generate a V_E_ of approximately 20 L/min/m^2^ body surface area (BSA) for each volunteer. There was a minimum of 4 days between exposures. Additional details related to volunteer characteristics, study design, facilities, measurement methods, exposure characterization, and results are available from Adams (2006).

The stated purpose of the Adams (2006) study was to compare the responses to the 0.08-ppm S-W exposure with the responses to the other five exposure conditions. Comparisons of responses among the other five exposure conditions (e.g., the 0.06-ppm S-W exposure with the FA exposure) at various time points were also presented. After the detection of statistically significant effects within the data using a two-way analysis of variance technique, the Scheffé multiple comparison test was used to identify which specific comparisons were significantly different. None of the differences between the FEV_1_ responses of exposure to 0.06 ppm O_3_ and FA were identified as statistically different from zero. However, Figure 1, which we adapted from Adams (2006), shows that the response to 0.06-ppm O_3_ exposure diverges over time from the response to FA. Additionally, the lack of overlap of the SE bars at the 6.6-hr time point suggests that the postexposure differences between FA and 0.06 ppm O_3_ are not likely to be attributed to chance alone.

Although the Scheffé method for detecting specific differences in the context of correcting for all possible contrasts in the data avoids type 1 statistical errors (false positives) at the level of the overall study, it is characterized as having relatively low power for detecting small differences for any single comparison of interest and is recognized as being a conservative test (Schwertman and Carter 1995). In the absence of calculations of statistical power or the probability of making a type 2 statistical error for the comparison between the postexposure responses of the FA and 0.06-ppm O_3_ exposures, we interpret the reported results of the Adams analysis as being inconclusive regarding the existence of an effect of 0.06-ppm O_3_ exposure on FEV_1_ response. That is, the inability to reject the null hypothesis of no difference between two conditions is not sufficient to conclude that no such difference exists.

Because of the potential regulatory implications of the presence or absence of effects at concentrations below the level of the current National Ambient Air Quality Standard (NAAQS) for O_3_ (currently 0.075 ppm) (NAAQS 2008), we elected to further examine the responses to 0.06 ppm O_3_ documented in the Adams (2006) study. Because the U.S. EPA risk assessment for lung function effects and the current NAAQS for O_3_ are generally based on the postexposure responses of a group of published 6.6-hr S-W studies, we conducted a targeted, secondary analysis of the Adams data to focus on the specific question of whether exposure to 0.06 ppm O_3_ for 6.6 hr results in FEV_1_ decrements relative to the FA exposure.

## Materials and Methods

The data presented here were originally collected for a recent study of humans exposed to low levels of O_3_ (Adams 2006). The exposures of interest for the purpose of the analysis presented here are the constant-concentration or S-W exposures to 0.06 ppm O_3_ and to FA. For purposes of comparison with other studies, we also present some results from the 0.08-ppm S-W O_3_ exposures from the Adams (2006) study. Although only group statistics were presented in the Adams (2006) publication, each subject’s FEV_1_ measurement before and after the 6.6-hr exposures was made available by the author to the U.S. EPA for inclusion in its Air Quality Criteria Document for O_3_ and Related Photochemical Oxidants (U.S. EPA 2006) and its health risk assessment (U.S. EPA 2007). We analyzed these individual pre- and postexposure FEV_1_ data.

The question of interest here is whether a 6.6-hr exposure to 0.06 ppm O_3_ under the conditions of the Adams (2006) study results in postexposure FEV_1_ decrements greater than those after a control exposure to FA. We calculated the FEV_1_ percent decrement for FA and for 0.06-ppm O_3_ exposures for each of the 30 participants as 100% × (preexposure FEV_1_ – postexposure FEV_1_)/preexposure FEV_1_. We then calculated the FEV_1_ response due to O_3_ (i.e., O_3_-induced) by subtracting the percent decrement after FA from the percent decrement after 0.06 ppm O_3_ for each volunteer. The distribution of O_3_-induced responses in these data did not appear to deviate markedly from a Gaussian distribution. However, it is well known that higher-dose O_3_ exposures that produce larger group mean FEV_1_ responses result in frequency distributions of response that are quite skewed, thereby potentially challenging some of the assumptions of many parametric statistical tests (Kulle et al. 1985; McDonnell 1996; Weinmann et al. 1995). The nonparametric sign test, which assumes only that the responses of each subject are independent and makes no assumptions about the distribution of the response data, is appropriate to test the null hypothesis that observed values have the same probability of being positive or negative (Fisher and Belle 1993). We therefore selected the sign test as our primary test of the null hypothesis that the FEV_1_ responses for the FA and 0.06-ppm exposures are not different.

Because the individual O_3_-induced decrements in this study were generally symmetrical and because other studies have demonstrated that exposures that produce mean responses of the magnitude observed in this study result in roughly symmetric response distributions (Kulle et al. 1985; McDonnell et al. 1983), we also compared the FA and 0.06-ppm O_3_ responses using other methods. We used the Wilcoxon signed rank test, which makes the assumption that the individual O_3_-induced responses are symmetrically distributed, and the paired *t*-test, which makes the further assumption that the responses are normally distributed (Fisher and Belle 1993).

We calculated two-sided statistical tests using SYSTAT 5.0 (Systat, Inc., Evanston, IL), and we examined Tukey box plots to assess potential outlier data using Graph Pad Prism 5 (Graph Pad Software, Inc., San Diego, CA).

## Results

Table 1 lists the individual percentage decrements in FEV_1_ from baseline after the FA and 0.06-ppm O_3_ exposures and the O_3_-induced decrements. For the full data set (*n* = 30), the O_3_-induced FEV_1_ responses appear to be symmetrically distributed around the median of 2.91% and mean of 2.85%. Twenty-four of the 30 individuals experienced an O_3_-induced decrement greater than zero (range, 0.23–14.52%), whereas six experienced an improvement in FEV_1_ (range, −0.43% to −7.42%) after the exposure. The null hypothesis that there is no difference between the median responses of the FA and the 0.06-ppm exposures (or that the median O_3_-induced decrement equals zero) is rejected using the two-tailed sign test (*p* = 0.0019). We also conducted alternative analyses of the data using the Wilcoxon signed rank test and the paired *t*-test. The null hypothesis of no difference between responses for the FA and 0.06-ppm O_3_ exposures was rejected by both tests (Table 2). Although the data had no extreme outliers, we flagged three responses as potential outliers according to the Tukey criteria. The characteristics of the data change little with exclusion of the single most extreme value (subject 23 in Table 1) or all three potential outliers (subjects 10, 12, and 23 in Table 1) removed (Table 2).

## Discussion

**Figure 1:**
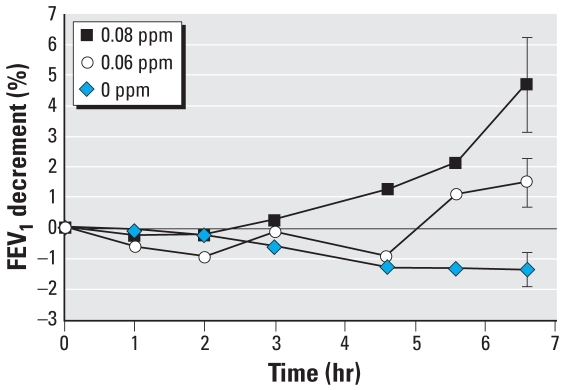
Mean FEV_1_ decrements as function of exposure duration and O_3_ concentration. Data are for constant, S-W O_3_ protocols in the Adams (2006) study. Error bars are the SE of responses at 6.6 hr. Adapted from Adams (2006).

**Figure 2:**
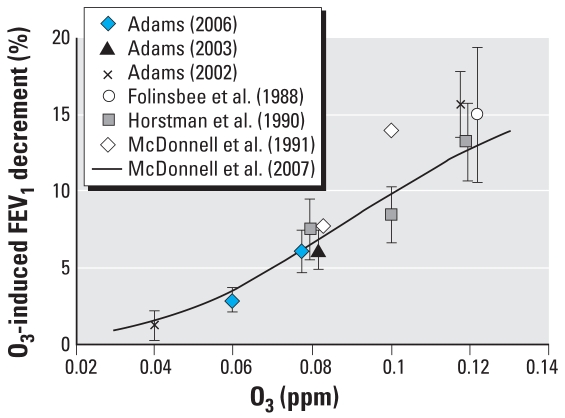
Cross-study comparison of mean O_3_-induced FEV_1_ decrements due to 6.6 hr of constant, S-W exposure to varied O_3_ concentrations. All exposures were conducted in a chamber, except for a face-mask exposure to 0.04 ppm O_3_ in the Adams (2002) study. All studies used a 6.6-hr exposure protocol in which volunteers alternated between 50 min of exercise (V_E_ ≈ 20 L/min/m^2^ BSA) and 10 min of rest with an additional 35 min of rest after the third hour. For this exposure protocol, the McDonnell et al. (2007) curve illustrates the predicted FEV_1_ decrement at 6.6 hr as a function of O_3_ concentration for a 23-year-old. Error bars (where available) are the SE of responses. The data at 0.08 and 0.12 ppm have been offset for illustrative purposes.

We have demonstrated that the FEV_1_ decrements that occur after a 6.6-hr exposure to 0.06 ppm O_3_ are statistically different from those that occur after FA exposure (*p* < 0.01). We arrive at the same finding regardless of the statistical test we employ or the approach to treating potential outliers in the data. That the effect of 0.06 ppm O_3_ on FEV_1_ occurred not by chance alone is further supported by two additional observations. First, the FA and 0.06-ppm responses in FEV_1_ generally track each other for the first 4.6 hr of exposure, with no evidence of wide swings in the data. The responses clearly diverge for both the 5.6- and 6.6-hr data points, indicating that the response at 6.6 hr is not a single anomalous data point (Figure 1). The group mean O_3_-induced decrement at 5.6 hr (~ 2.4%) is only marginally smaller than that at 6.6 hr (2.85%). This temporal pattern of response is generally consistent with patterns of response after 0.08 ppm, 0.10 ppm, and 0.12 ppm in numerous studies in which the O_3_ response begins to diverge from the FA response at earlier time points during exposure to higher concentrations of O_3_ (Adams 2000, 2002, 2003; Folinsbee et al. 1988; Horstman et al. 1990). Second, the magnitude of the group mean O_3_-induced response of this sample after exposure to 0.08 ppm O_3_ is 6.07%, which is quite consistent with observed responses to 0.08-ppm exposure from other studies (Figure 2), indicating that this sample of volunteers is not unusually sensitive to O_3_. We thus have no reason to suspect that the observed average response at 0.06 ppm is inflated because of attributes of the target population or sampling error.

The existence of a small group mean FEV_1_ decrement after a 6.6-hr exposure of young exercising adults to 0.06 ppm should not come as a surprise. Figure 2 presents response data from studies that used an exposure protocol nearly identical to that used by Adams (2006). These studies all used young, healthy adults as volunteers; exposure duration was for 6.6 hr, and exercise pattern and V_E_ (~20 L/min/m^2^ BSA) were similar. The group mean FEV_1_ response to the 0.08-ppm exposure for the Adams (2006) study is consistent with other studies. Furthermore, the observed FEV_1_ responses at 0.04 ppm O_3_ (Adams 2002) and 0.06 ppm O_3_ (Adams 2006) are almost identical to the response predicted by a model based on data from 15 studies conducted at the U.S. EPA Human Studies Facility in Chapel Hill, North Carolina (McDonnell et al. 2007). The smooth C–R curve illustrated in Figure 2 is consistent with C–R curves for shorter-duration (2 hr) exposures, which have typically been observed to be smooth without obvious discontinuities over a wide range of concentrations, including those where effects were near zero (Avol et al. 1984; Hazucha 1987; Kulle et al. 1985; McDonnell et al. 1983).

The stark difference between our conclusions and those of Adams (2006) with regard to whether a 6.6-hr exposure to 0.06 ppm O_3_ induces statistically significant FEV_1_ decrements requires further explanation. We have identified three factors that we believe contribute to the difference in conclusions. First, no *p*-value is given in the original manuscript for the comparison of the 6.6-hr FA and 0.06-ppm responses. The group mean difference of 2.85% is simply reported as not being statistically significantly different from zero at α = 0.05 using the Scheffé test to correct for the multiple comparisons. However, in Table 3 of the original manuscript, the difference between the 0.08-ppm and 0.06-ppm exposures after 6.6 hr (3.21%) is reported as being statistically different from zero. Because the analysis of variance and the Scheffé method used by Adams assume equal variances for all means, it is reasonable to assume that the difference of 2.85% between 0.06 ppm and FA approached statistical significance—even with this most conservative methodology.

A second factor causing differences in conclusions reached herein compared with those of Adams (2006) results from differences in the purpose of our reanalysis compared with the purpose of the original study, the statistical approaches used, and the approach to controlling for multiple statistical tests. The stated purpose of the Adams (2006) study implies a large number of comparisons among six exposure protocols and seven time points (0, 1, 2, 3, 4.6, 5.6, and 6.6 hr). The Scheffé method allows all pairwise comparisons as well as an infinite number of linear contrasts among the cell means to be made while limiting the probability of making a single type 1 statistical error among all the potential contrasts to 0.05. Although there is nothing inherently wrong with making a large number of contrasts and strictly maintaining a studywide alpha level using the Scheffé method, this approach comes at the cost of reduced statistical power for assessing differences for specific comparisons. Setting aside all the theoretically possible linear contrasts, there are 15 possible direct comparisons between the six protocols used by Adams (2006) at the 6.6-hr time point. The Bonferroni procedure would be the preferred multiple comparison correction for this relatively small number of comparisons (Schwertman and Carter 1995). A critical alpha for the possible comparisons between the six protocols at the 6.6-hr time point would be 0.05 ÷ 15, or 0.0033. We compared only the FEV_1_ responses at 6.6 hr between FA and 0.06-ppm O_3_ S-W exposure. Even with a correction for the additional comparisons, which we did not consider, all of the tests presented in Table 2 remain statistically significant.

The third factor potentially contributing to the disparity in our conclusions compared with those of Adams (2006) involves errors caused by data not meeting the criteria for the statistical tests applied. The two-factor analysis of variance and Scheffé method cited by Adams (2006) assume that data are normally distributed and that variances are equal within cells. From previous studies of higher O_3_ exposures, we know that FEV_1_ responses become skewed and that variance increases (McDonnell 1996). This increase in variance is clearly apparent in the Adams (2006) data, with the standard deviation of FEV_1_ responses increasing from 2.98% for FA to 4.24% for 0.06 ppm O_3_ to 8.65% at 0.08 ppm. It is possible that the wide range in variances among the cells resulted in wider confidence levels and inaccuracies of the reported *p*-values for a subset of the comparisons.

Assuming for the moment that the O_3_-induced decrement at 0.06 ppm is not zero and that the best point estimate of the magnitude of the mean decrement is 2.85% (95% confidence interval, 1.26–4.45%), what can we say about the possible clinical significance of reversible effects of such magnitude? It is unlikely that individuals experiencing a 3% reduction in FEV_1_ would be aware of such an effect, and effects of this magnitude are at the outer range of expected day-to-day variability of the measurement. We know, however, that individuals vary reproducibly in their responses to O_3_ exposure, and it is those most responsive individuals who are likely to experience clinically significant effects. Although the average response was small, two subjects in the Adams (2006) study had > 10% FEV_1_ decrements (12.8% and 14.5%). The U.S. EPA has considered any individual changes in FEV_1_ of 10–20% to be a moderate effect clearly outside the range of normal within-day variability (U.S. EPA 2006, 2007). In the Adams (2006) study, 2 of 30 individuals (i.e., 7%) experienced such effects. This observed proportion of individuals having a > 10% decrement in FEV_1_ at 0.06 ppm is consistent with a smooth C–R curve that includes observed proportions from previous studies of 26% at 0.08 ppm, 41% at 0.10, and 57% at 0.12 ppm [proportions calculated for S-W exposures in U.S. EPA (2007), Table 5–3]. If one converts the SD values to SE values in Adams (2006, Table 4), the SEs for the symptoms resulting from the 0.06-ppm S-W exposure do not overlap those for the FA exposure. This is suggestive of an effect of 0.06 ppm on respiratory symptoms as well as FEV_1_. A reversible loss of lung function in combination with the presence of symptoms is considered adverse (American Thoracic Society 2000). It thus appears that a small fraction of individuals exposed to 0.06 ppm O_3_ for 6.6 hr during moderate exercise may approach a degree of response that is considered to be adverse.

To meet the objectives of his study, Adams (2006) required that a large number of comparisons be made and that comparisons not be identified as statistically significant by chance alone. Appropriately, the Scheffé test was employed to meet these needs. Although Adams (2006) did not find the FEV_1_ responses of the 0.06 ppm O_3_ and FA exposures to be statistically different, no measure of the probability of a type 2 statistical error was provided, which would be required before drawing a conclusion that a true difference does not exist. The objective of the present study was to reevaluate the lung function data of subjects exposed for 6.6 hr to 0.06 ppm O_3_. Regardless of the statistical test used, we found the post-exposure FEV_1_ decrements for the FA and 0.06-ppm O_3_ exposures to be strongly significantly different. Complete resolution of this question must necessarily await further studies designed to address this specific question with adequate statistical power. However, comparison with the results of other studies support the interpretation that our finding is not due to chance alone and, indeed, is consistent with a smooth C–R curve.

## Figures and Tables

**Table 1:** Percent decrement in FEV_1_ for 6.6 hr of exposure to FA and 0.06 ppm O_3_ for individuals in the Adams (2006) study.

Subject	ΔFA	ΔO_3_	ΔO_3_ – ΔFA
1	−1.46	−0.87	0.59
2	0.20	3.01	2.81
3	−0.29	5.93	6.23
4	−5.33	−3.52	1.81
5	−8.62	−3.75	4.87
6	−2.94	−3.89	−0.95
7	−0.21	0.21	0.42
8	−0.28	3.43	3.71
9	−3.12	3.91	7.03
10	−6.32	6.43	12.76^a^
11	−0.53	0.53	1.07
12	4.76	−2.66	−7.42^a^
13	−1.71	−2.87	−1.16
14	−0.42	2.87	3.29
15	−1.16	−0.57	0.58
16	−5.54	−1.74	3.80
17	−0.62	−1.05	−0.43
18	−4.35	1.88	6.23
19	−3.17	1.98	5.15
20	2.02	5.03	3.00
21	−2.19	3.66	5.85
22	−2.19	−5.13	−2.93
23	0.21	14.73	14.52^a^
24	−0.58	0.59	1.17
25	−0.27	0.54	0.81
26	−4.14	1.10	5.24
27	1.79	2.02	0.23
28	0.19	−0.40	−0.60
29	0.87	4.21	3.34
30	5.02	9.62	4.60
Mean	−1.35	1.51	2.85
SD	2.98	4.24	4.28

ΔO_3_ – ΔFA represents the decrements due to 0.06 ppm O_3_ corrected for any FA effects. Data are FEV_1_ percent decrements from preexposure; a negative number indicates an increase or improvement in FEV_1_.

a Potential outlier according to the Tukey criteria.

**Table 2:** Descriptive and inferential statistics for O_3_-induced decrements^a^ in FEV_1_ for the full data set (*n* = 30) and for two data sets with potential outliers removed.

	Data set		
	*n* = 30	*n* = 29	*n* = 27
Median^a^	2.91%	2.81%	2.81%
Mean^a^	2.85%	2.45%	2.44%
SE^a^	0.78%	0.69%	0.51%
95% confidence interval^a^	1.26–4.45%	1.03–3.87%	1.38–3.49%
Sign test *p*-value^b^	0.0019	0.0030	0.0021
Wilcoxon *p*-value^b^,^c^	0.0008	0.0014	0.0004
Paired *t*-test *p*-value^b^	0.0010	0.0014	<0.0001

a O_3_-induced decrements are the difference between percent decrements in FEV_1_ from the preexposure for the 0.06 ppm O_3_ and FA exposures.

b *p*-Values are two sided.

c Wilcoxon signed rank test.
